# LRP1 modulates the microglial immune response via regulation of JNK and NF-κB signaling pathways

**DOI:** 10.1186/s12974-016-0772-7

**Published:** 2016-12-08

**Authors:** Longyu Yang, Chia-Chen Liu, Honghua Zheng, Takahisa Kanekiyo, Yuka Atagi, Lin Jia, Daxin Wang, Aurelie N’songo, Dan Can, Huaxi Xu, Xiao-Fen Chen, Guojun Bu

**Affiliations:** 1 Institute of Neuroscience, Fujian Provincial Key Laboratory of Neurodegenerative Disease and Aging Research, Medical College, Xiamen University, Xiamen, 361102 China; 2 Department of Neuroscience, Mayo Clinic, 4500 San Pablo Road, Jacksonville, FL32224 USA; 3 Shenzhen Research Institute of Xiamen University, Shenzhen, 518063 China

**Keywords:** LRP1, Microglia, Inflammation, JNK, NF-κB, RAP, AD

## Abstract

**Background:**

Neuroinflammation is characterized by microglial activation and the increased levels of cytokines and chemokines in the central nervous system (CNS). Recent evidence has implicated both beneficial and toxic roles of microglia when over-activated upon nerve injury or in neurodegenerative diseases, including Alzheimer’s disease (AD). The low-density lipoprotein receptor-related protein 1 (LRP1) is a major receptor for apolipoprotein E (apoE) and amyloid-β (Aβ), which play critical roles in AD pathogenesis. LRP1 regulates inflammatory responses in peripheral tissues by modulating the release of inflammatory cytokines and phagocytosis. However, the roles of LRP1 in brain innate immunity and neuroinflammation remain unclear.

**Methods:**

In this study, we determined whether LRP1 modulates microglial activation by knocking down *Lrp1* in mouse primary microglia. LRP1-related functions in microglia were also assessed in the presence of LRP1 antagonist, the receptor-associated protein (RAP). The effects on the production of inflammatory cytokines were measured by quantitative real-time PCR (qRT-PCR) and enzyme-linked immunosorbent assay (ELISA). Potential involvement of specific signaling pathways in LRP1-regulated functions including mitogen-activated protein kinases (MAPKs) and nuclear factor-κB (NF-κB) were assessed using specific inhibitors.

**Results:**

We found that knocking down of *Lrp1* in mouse primary microglia led to the activation of both c-Jun N-terminal kinase (JNK) and NF-κB pathways with corresponding enhanced sensitivity to lipopolysaccharide (LPS) in the production of pro-inflammatory cytokines. Similar effects were observed when microglia were treated with LRP1 antagonist RAP. In addition, treatment with pro-inflammatory stimuli suppressed *Lrp1* expression in microglia. Interestingly, NF-κB inhibitor not only suppressed the production of cytokines induced by the knockdown of *Lrp1* but also restored the down-regulated expression of *Lrp1* by LPS.

**Conclusions:**

Our study uncovers that LRP1 suppresses microglial activation by modulating JNK and NF-κB signaling pathways. Given that dysregulation of LRP1 has been associated with AD pathogenesis, our work reveals a critical regulatory mechanism of microglial activation by LRP1 that could be associated with other AD-related pathways thus further nominating LRP1 as a potential disease-modifying target for the treatment of AD.

## Background

Microglia are the resident innate immune cells in the central nervous system (CNS) ubiquitously distributed in the brain [1]. When severe injury occurs, microglia change their morphology and migrate to the lesion sites. They proliferate and phagocytize dying cells and other debris and/or release cytokines to maintain the homeostasis of microenvironment impacting neuronal function and survival [2]. However, mounting evidence has also implicated the neurotoxic roles of microglia when they are over/chronically activated in neurodegenerative diseases or under conditions of severe injury [3]. Microglia have been widely studied for their roles in Alzheimer’s disease (AD) pathogenesis [4–7]. Microglia activated by amyloid-β (Aβ) in vitro exhibit increased expression of pro-inflammatory cytokines, including interleukin-1β (IL-1β), tumor necrosis factor-α (TNF-α), IL-6, and IL-8, that cause neuronal damage [8]. Recent genetic studies have identified several inflammation-related genes in macrophage/microglia, including *TREM2*, *CD33*, *CR1*, and *ABCA7*, that linked to the risk of late-onset AD (LOAD) [9–11]. As aberrant activation or impaired function of the innate immune system contributes to the pathological initiation and propagation of AD [12, 13], dissecting the molecular mechanism underlying microglial activation would be beneficial for AD drug development and therapy.

The low-density lipoprotein receptor-related protein 1 (LRP1) is a type I transmembrane glycosylated protein that consists of the 515-kDa extracellular α-chain coupled to the cell surface through non-covalent interaction with the transmembrane 85-kDa β-chain [14, 15]. In the CNS, LRP1 is ubiquitously expressed and serves as a critical transport receptor as well as a modulator of several distinct signaling pathways in the vasculature [16, 17], blood brain barrier [18], neurons [19], astrocytes [20], and microglia [21]. LRP1 regulates the metabolism of over 40 ligands, including Aβ and apolipoprotein E (apoE) as well as proteases and growth factors implicated in inflammation [14, 22, 23]. Conditional deletion of the mouse *Lrp1* gene in forebrain neurons leads to an increase in glial activation and elevated production of pro-inflammatory cytokines [24]. Deficiency of LRP1 in macrophage leads to down-regulation of anti-inflammatory markers while enhancing the macrophage response to pro-inflammatory stimuli [25]. In the peripheral nervous system, soluble LRP1 (sLRP1), which consists of the entire LRP1 α-chain and part of the β-chain ectodomain, can bind directly to Schwann cell surfaces and inhibit the cellular response to TNF-α [26]. It has also been demonstrated that LRP1 intracellular domain (LICD) suppresses lipopolysaccharide (LPS)-induced inflammatory responses by binding to the interferon-γ promoter in macrophage [27]. In addition, activation of the LDL receptor family members has been reported to modulate glial inflammation by modulating mitogen-activated protein kinase [28]. However, the molecular mechanism underlying LRP1-mediated inflammation in CNS remains unclear. In this study, we investigated whether and how LRP1 mediates microglial activation and further unraveled the signaling pathways underlying LRP1 functions in microglia.

## Methods

### Antibodies and chemical reagents

The following antibodies were used in this study: anti-MAP2 (Cell Signaling), anti-GFAP (Abcam), anti-Iba-1 (Wako), anti-apoE (Meridian Life Science), anti-Phospho-SAPK/JNK (Thr183/Tyr185), anti-JNK, anti-c-Jun, anti-Phospho-c-Jun (Ser73), anti-NF-κB p65, anti-Phospho-NF-κB p65 (Ser536), anti-Phospho-p44/42 MAPK (Erk1/2) (Thr202/Tyr204), anti-p44/42 MAPK (Erk1/2), anti-p38 MAPK, anti-Phospho-p38 MAPK, anti-Phospho-IκBα (Ser32), anti-IκBα, and anti-β-actin (Cell Signaling). Rabbit polyclonal anti-LRP1 was produced in our laboratory [29]. LPS, mouse TNF-α, NF-κB inhibitor (BAY 11-7082), and JNK inhibitor (SP600125) were purchased from Sigma-Aldrich.

Oligomeric Aβ42 was obtained from the Proteomics Core at the Mayo Clinic and prepared as previously described [30]. Briefly, aliquots of 100 μM Aβ monomer purified by size exclusion chromatography were incubated overnight at room temperature in 50 mM NaCl and 4 mM SDS. To remove SDS and reduce salt concentration, the sample was dialyzed against 20 mM sodium phosphate buffer at pH 7.0 (NaP) for 48–72 h and then against 10 mM NaP. Sample quality was monitored and confirmed at each step of the preparation by circular dichroism (CD) and thioflavin T fluorescence. Residual or unconverted monomer was removed by filtering the dialyzed oligomer with an Amicon Ultra 4 centrifugal concentration/filtration device with a MW cutoff of 50 kDa.

### Expression and purification of recombinant RAP

Recombinant receptor-associated protein (RAP) was purified as described previously [31] with minor modifications. Briefly, DH5α bacteria harboring the GST-RAP protein were grown at 37 °C to an O.D. of 0.7 at 600 nm. Expression was induced by the addition of isopropylthio-β-d-galactoside to a final concentration of 0.01%, and the cultures were grown for another 4 h at 30 °C. Bacteria were harvested by centrifugation at 4 °C and resuspended in PBS containing 1% (*v*/*v*) Triton X-100, 1 mM PMSF, complete proteinase inhibitor, 1 mM EDTA. Bacteria were sonicated and centrifuged at 26,000*g* for 30 min at 4 °C. The supernatant was mixed with glutathione beads at 4 °C, washed in PBS, and thereafter with 50 mM Tris-HCl at pH 8.0. Bound GST-RAP protein was eluted with 50 mM Tris-HCl containing 20 mM reduced glutathione at pH 8.0. The eluate was dialyzed against 50 mM Tris-HCl at pH 8.0, and the fusion protein was cleaved with thrombin in 50 mM Tris-HCl, 150 mM NaCl, and 2.5 mM CaCl2 at pH 8.0. The free RAP was removed from the GST via heparin sepharose column. Following washes with 20 mM Tris-HCl at pH 7.4, RAP was eluted from the column with 20 mM Tris-HCl, 2 M NaCl at pH 7.4, and was then dialyzed against 50 mM Tris-HCl at pH 7.4. To remove the potential bacterial endotoxin, purified RAP was incubated with endotoxin removal resin (Thermo Fisher) according to the manufacturer’s instruction. Before treatment, RAP stock solutions were filtered with 0.22 μm sterile syringe filters.

### Mouse primary cell culture

Primary microglia were prepared as previously described [32, 33] with minor modifications. Briefly, mixed glial cells from C57BL/6J neonatal mice at postnatal day 1 to day 3 (P1-P3) were cultured in DMEM (Gibco) supplemented with 10% heat-inactivated FBS (Moregate) and 1% penicillin streptomycin solution (100 U/mL penicillin, and 100 μg/mL streptomycin, Invitrogen). The medium was changed 3 days later with fresh DMEM medium containing 10% FBS, 1% penicillin streptomycin, and 25 ng/mL granulocyte-macrophage colony-stimulating factor (GM-CSF, R&D Systems). Primary microglia were harvested by shaking after 10–12 days in culture and once a week thereafter for up to three times. The purity of the isolated microglia was >95% as determined by flow cytometry analysis with antibody against CD11b (BD Biosciences).

### *Lrp1* knockdown by small-interfering RNA

Mouse *Lrp1*-specific siRNAs and non-targeting control were purchased from Dharmacon Research. The siRNA sequences for mouse *Lrp1* were as followed: *Lrp1* siRNA1: 5′-GGAGUCACUUACAUCAAUAUU-3′; *Lrp1* siRNA2: 5′-GCAGCGAGCCAACAAG UAU-3′. siRNAs were transfected into primary microglia using Basic Nucleofector™ Kit for Primary Mammalian Glial Cells (VPI-1006, Lonza) according to the manufacturer’s specifications. Each electroporation reaction contained 6 × 10^6^ cells and 300 nM siRNA. Cells were further cultured for 48 h before using in experiments and analysis.

### Primary microglia treatment

Primary microglia from C57BL/6J neonatal mice was cultured in the medium without GM-CSF for 24 h. Cells were then transferred to serum-free medium for 30 min and then treated with RAP (25 or 50 nM) for 4 h (for quantitative real-time PC (qRT-PCR)) or 30 min (for Western blotting). Cells were treated with LPS (100 ng/ml) as a positive control. For the treatment with inflammatory stimuli, primary microglia was cultured in the medium without GM-CSF for 24 h. Cells were further transferred to serum-free medium for 30 min and then cultured in the presence of either PBS (control), LPS (100 ng/ml), mouse TNF-α (100 ng/ml), or oligomeric Aβ (10 μM) for another 24 h (for qRT-PCR and Western blotting).

### Western blotting

Cells were harvested and homogenized in Nonidet P-40 lysis buffer (1% Nonidet P-40, 50 mM Tris-HCl, pH 8.0, 150 mM sodium chloride supplemented with protease inhibitors cocktail). The samples were centrifuged at 12,000×*g* for 15 min at 4 °C. The supernatant was collected, and total protein levels were measured by the micro-BCA protein assay kit (Thermo Fisher Scientific). Equal amounts of total proteins were separated by SDS-PAGE and transferred to PVDF membrane (Millipore). The membranes were blocked with 5% nonfat milk in TBST (Tris-buffered saline, 20 mM Tris-HCl, 137 mM NaCl, 0.1% Tween-20, pH 7.6) and probed with primary antibodies, followed by treatment with HRP-linked secondary antibodies and ECL Western blotting detection reagents. The intensity of immune-reactive bands was quantified using ImageJ software.

### Quantitative real-time PCR

Total RNA was isolated from cells using TRIzol reagent (Invitrogen) and then dissolved in nuclease-free water and stored at −80 °C. Reverse transcription was performed using a ReverTra Ace qPCR RT Master Mix with gDNA Remover (TOYOBO) according to the manufacturer’s protocol. Quantitative real-time PCR (qRT-PCR) was performed using the FastStart Universal SYBR Green Master (Roche) on the 7500 fast real-time PCR platform (ABI). Each reaction was run in triplicate, and the real-time value for each sample was averaged and compared using the CT method, where the amount of target RNA (2^−ΔΔCT^) was obtained by normalization to an endogenous reference (β-actin) and relative to a calibrator. Gene expression was considered undetectable if the Ct value was greater than 33 cycles. The primer sequences for *Lrp1*, *Il-1β*, *Tnf-α*, *Il-10*, *Apoe*, and *β-actin* were as follows: *Lrp1*-forward: 5′-ACTATGGATGCCCCTAAAACTTG-3′; *Lrp1*-reverse: 5′-GCAATCTCTTTCACCGTCACA-3′; *Il-1β*-forward: 5′-GCAACTGT TCCTGAACTCAACT-3′; *Il-1β*-reverse: 5′-ATCTTTTGGGGTCCGTCAACT-3′; *Tnf-α*-forward: 5′-CCCTCACACTCAGATCATCTTCT-3′; *Tnf-α*-reverse: 5′-GCTACGAC GTGGGCTACAG-3′; *Il-10*-forward: 5′-GCTCTTACTGACTGGCATGAG-3′; *Il-10*-reverse: 5′-CGCAGCTCTAGGAGCATGTG-3′; *Apoe*-forward: 5′-CTGACAGGATGC CTAGCCG-3′; *Apoe*-reverse: 5′-CGCAGGTAATCCCAGAAGC-3′; *β-actin*-forward: 5′-AGTGTGACGTTGACATCCGTA-3′; *β-actin*-reverse: 5′-GCCAGAGCAGTAATC TCCTTC.

### Cytokine ELISA

IL-1β and TNF-*α* in conditioned media were measured using the antibodies and reference standards contained in R&D Systems DuoSet enzyme-linked immunosorbent assay (ELISA) kits according to the manufacturer’s protocol.

### Statistical analysis

Statistical analysis was performed using GraphPad Prism 5.0 (GraphPad Software). Data were presented as average ± SEM. Data were analyzed by one-way ANOVA followed by Tukey’s post hoc analysis. All experiments were repeated with a minimum of three times. A *p* value of <0.05 was considered as statistically significant.

## Results

### Knockdown of *Lrp1* exacerbates LPS-stimulated pro-inflammatory cytokine production

**Fig. 1:**
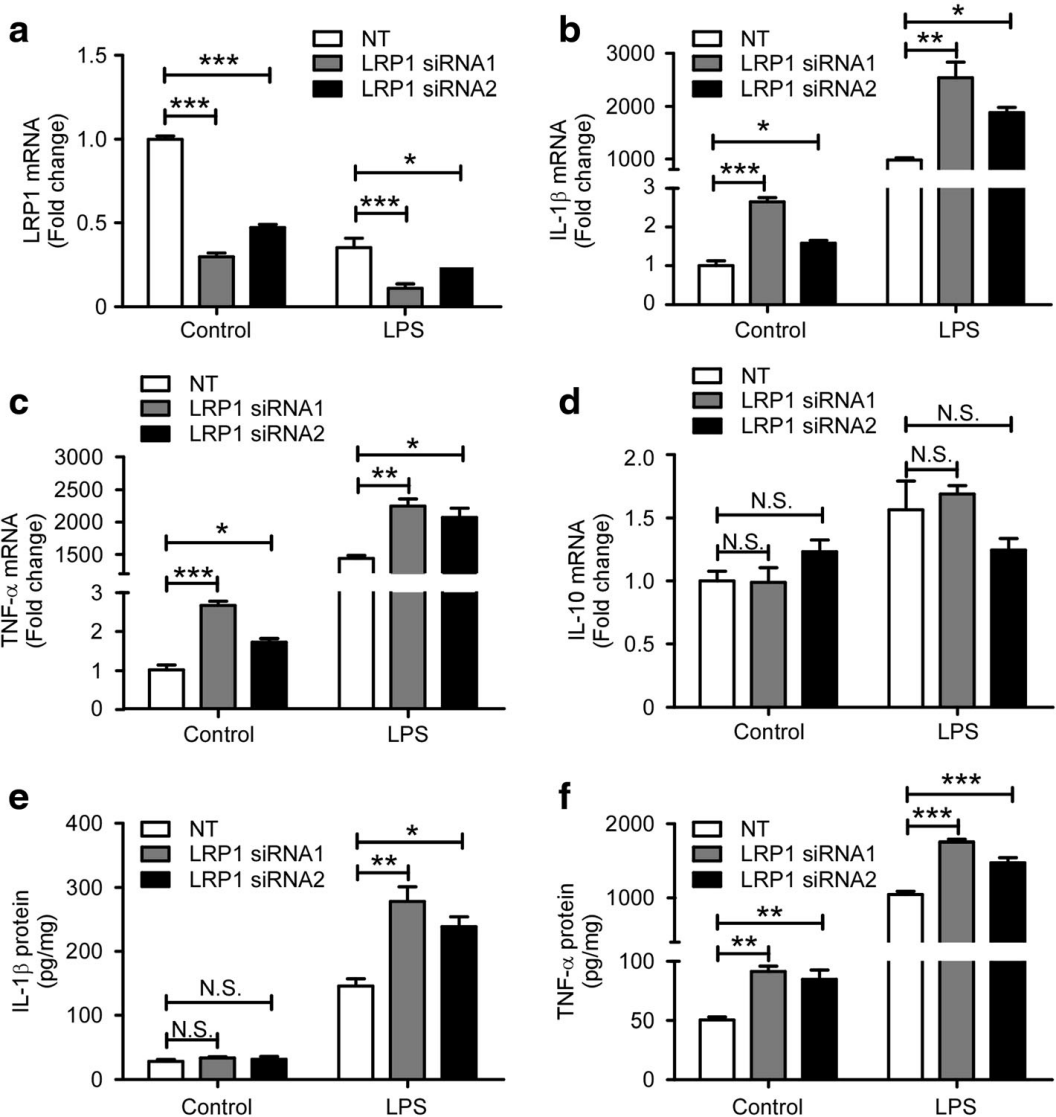
*Lrp1* knockdown exacerbates the production of pro-inflammatory cytokines. **a**–**d** Primary microglia were transiently transfected with non-targeting siRNA (NT) or two independent *Lrp1-*specific siRNAs for 48 h, then incubated with serum-free medium in the presence or absence of LPS (100 ng/ml) for 4 h (for qRT-PCR) or 24 h (for ELISA). RNA was extracted and the relative mRNA levels of *Lrp1* (**a**), *Il-1β* (**b**), *Tnf-α* (**c**), and *Il-10* (**d**) were determined by qRT-PCR and shown as bar graph (*n* = 3). β-Actin was used as an internal control. IL-1β (**e**) and TNF-α (**f**) in conditioned media were determined by ELISA (*n* = 3). Data were plotted as mean ± SEM and normalized to the corresponding control group. *p < 0.05; **p < 0.01; ***p < 0.001 (one-way ANOVA with post hoc Tukey’s *t* test)

Previous studies have demonstrated a role for LRP1 in the inflammatory responses in macrophage [25–27]. To evaluate the impact of LRP1 on the production of inflammatory cytokines in microglia, we established experimental conditions under which *Lrp1* was knocked down in mouse primary microglia using specific LRP1-siRNAs. By using two independent siRNAs (*Lrp1*-siRNA1 and *Lrp1*-siRNA2) targeting distinct regions of *Lrp1*, LRP1 expression level was successfully knocked down to 25 and 50%, respectively, compared with control non-targeting siRNA (NT) (Fig. 1a). We then measured the expression levels of the pro-inflammatory cytokines IL-1β and TNF-α, and anti-inflammatory cytokines IL-10 with or without microglial exposure to 100 ng/mL LPS for 4 h. Compared with the control siRNA treatment, we found that the mRNA levels of pro-inflammatory cytokines IL-1β and TNF-α were significantly increased in *Lrp1*-knockdown (*Lrp1*-KD) microglia both in the presence or absence of LPS (Fig. 1b, c). No obvious differences were observed for the level of anti-inflammatory cytokine IL-10 (Fig. 1d). Moreover, the protein levels of IL-1β and TNF-α in the media were increased in *Lrp1*-KD microglia compared with those of the control group as measured by ELISA (Fig. 1e, f). Treatment with the two independent *Lrp1* siRNAs both increased the levels of pro-inflammatory cytokines, suggesting that such effects are specific rather than a potential off-target effect.

### JNK and NF-κB pathways are activated in *Lrp1*-knockdown primary microglia

**Fig. 2:**
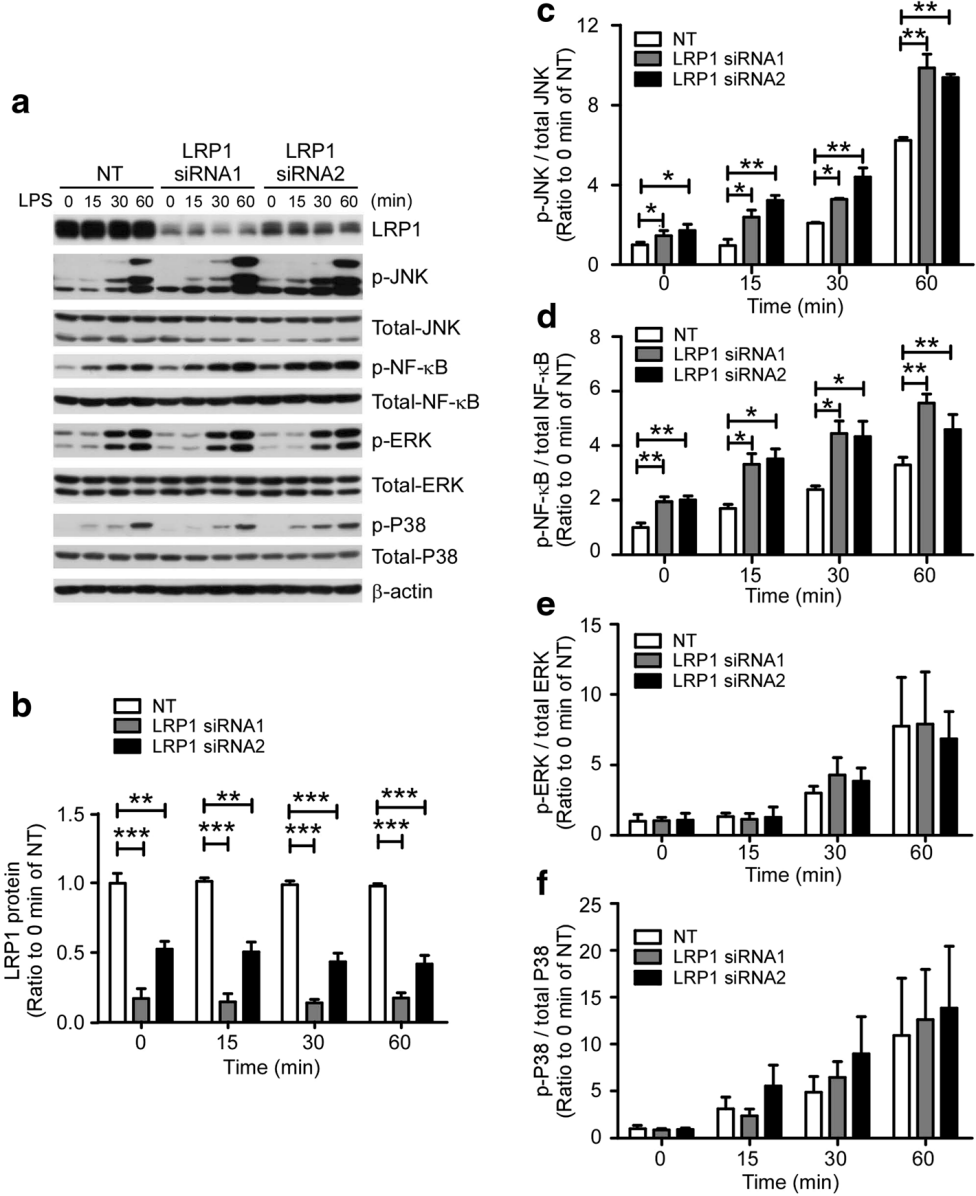
Both JNK and NF-κB pathways are activated in *Lrp1*-deficient primary microglia. **a** Primary microglia were transiently transfected with non-targeting siRNA (NT) or *Lrp1*-specific siRNAs for 48 h. Cells were stimulated with 100 ng/mL LPS for the indicated time (min). Cell lysates were prepared and analyzed by Western blotting using antibodies specific for LRP1, NF-κB, JNK, p38, ERK, or the phosphorylated forms of these proteins. β-Actin served as a loading control. **b**–**f** The quantification of Western blots of LRP1 (**b**), ratios of phospho-JNK/total JNK (**c**), phospho-NF-κB/total NF-κB (**d**), phospho-ERK/total ERK (**e**), and phospho-p38/total p38 (**f**). The relative signal intensities of each pathway at various time points were normalized to NT-treated microglia in the absence of LPS stimulation (0 min time point) (*n* = 3). Data were plotted as mean ± SEM *p < 0.05; **p < 0.01; ***p < 0.001 (one-way ANOVA with post hoc Tukey’s *t* test)

To explore the potential mechanisms through which LRP1 regulates LPS-induced cytokine production, we examined the activation kinetics of the three mitogen-activated protein kinase (MAPK) pathways (p38-MAPK, ERK, and JNK) and the NF-κB pathway, which are reported to function downstream of LPS stimulation [34]. For these experiments, cells were treated with 100 ng/mL LPS for 15, 30, and 60 min (Fig. 2a, b). Intriguingly, the phosphorylation of JNK was significantly higher in *Lrp1*-KD microglia compared with control cells before LPS stimulation and further enhanced upon LPS treatment (Fig. 2a, c). Additionally, the levels of phosphorylated NF-κB were also significantly higher in *Lrp1*-KD microglia than in control cells without LPS treatment, and the trend persisted for an extended period of time upon LPS stimulation (Fig. 2a, d). However, the phosphorylation kinetics of ERK and p38-MAPK were similar in control and *Lrp1*-KD microglia (Fig. 2a, e, f). Together, these results indicated that LRP1 modulates both JNK and NF-κB signaling pathways in microglial cells.

### LRP1 antagonist RAP regulates inflammation in microglia by modulating JNK and NF-κB pathways

**Fig. 3:**
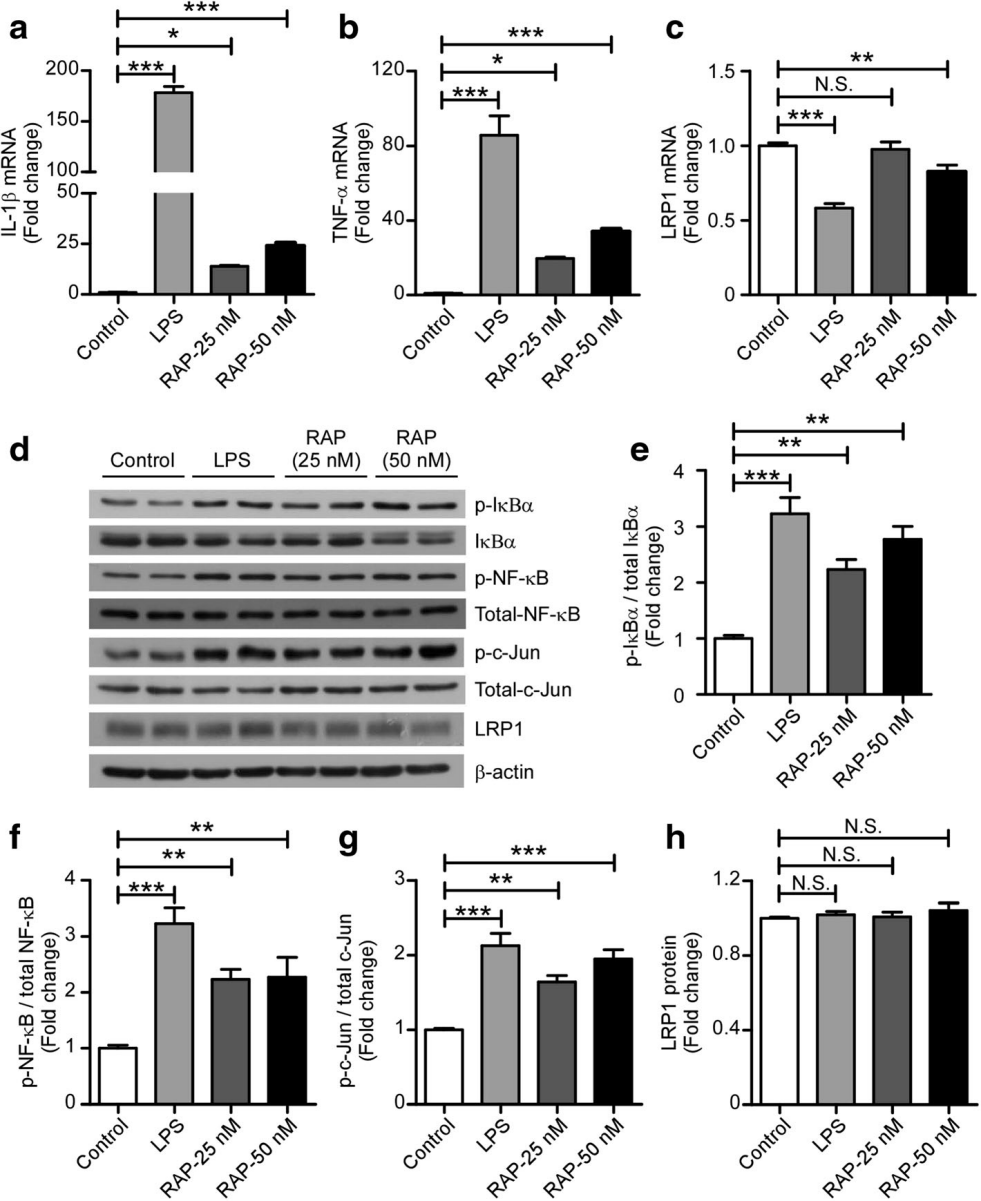
RAP increases the expression of pro-inflammatory cytokines and induces both JNK and NF-κB activation in microglia. **a**–**c** Primary microglia were treated with control, LPS (100 ng/ml) or RAP (25 or 50 nM) for 4 h (for qRT-PCR) or 30 min (for Western blotting). The qRT-PCR was performed to determine mRNA levels for *Il-1β* (**a**), *Tnf-α* (**b**), and *Lrp1* (**c**) (*n* = 3). **d**–**h** The protein levels of phospho-IκBα, total IκBα, phospho-NF-κB, total NF-κB, phospho-c-Jun, total c-Jun, and LRP1 in cell lysates were examined by Western blot analysis (**d**) and quantified (**e**–**h**) (*n* = 3). β-Actin served as a loading control. Data were plotted as mean ± SEM and normalized to the corresponding control group. *p < 0.05; **p < 0.01; ***p < 0.001; *N.S*. not significant (one-way ANOVA with post hoc Tukey’s *t* test)

LRP1 interacts with a variety of ligands involved in cell signaling [22]. Receptor-associated protein (RAP) is a specialized endoplasmic reticulum (ER) chaperone for LDL receptor family members, including LRP1 [35]. RAP functions in receptor folding and trafficking by blocking premature ligand binding during receptor maturation [36]. RAP binds to LRP1 with high affinity and has been widely used as an antagonist for LRP1 [37, 38]. To further examine the role of LRP1 in the inflammatory responses of microglia, we treated primary microglia with LPS (100 ng/mL) or RAP (25 and 50 nM) for 4 h. LPS in these experiments served as a positive control for cytokine production and microglial activation. Our results showed that RAP increased the expression of IL-1β and TNF-α in a dose-dependent manner (Fig. 3a, b). In addition, modest but significant change in *Lrp1* mRNA was observed in cells treated for 4 h with high concentration of RAP (50 nM), suggesting that higher concentration of RAP suppress the expression of *Lrp1* in microglia (Fig. 3c). We further examined the effects of RAP on the inflammation-related signaling pathways in microglia. Upon treatment of primary microglia with LPS or RAP (25 and 50 nM) for 30 min, we observed increased phosphorylation of IκBα and NF-κB, suggesting that LRP1 inhibition leads to NF-κB activation (Fig. 3d–f). RAP also induced phosphorylation of c-Jun indicating an activation of JNK signaling pathway (Fig. 3d, g). In particular, we found that LRP1 protein level was unchanged after short time (30 min) treatment with LPS or RAP (Fig. 3h), indicating that the enhanced inflammatory responses here are due to direct effects on LRP1 functional inhibition rather than changes in LRP1 expression. These results indicated that LRP1 antagonist RAP can increase the production of pro-inflammatory cytokines by activating JNK and NF-κB signaling pathways.

### JNK and NF-κB inhibitors eliminate the hypersensitivity of LRP1-knocked down microglia to LPS

**Fig. 4:**
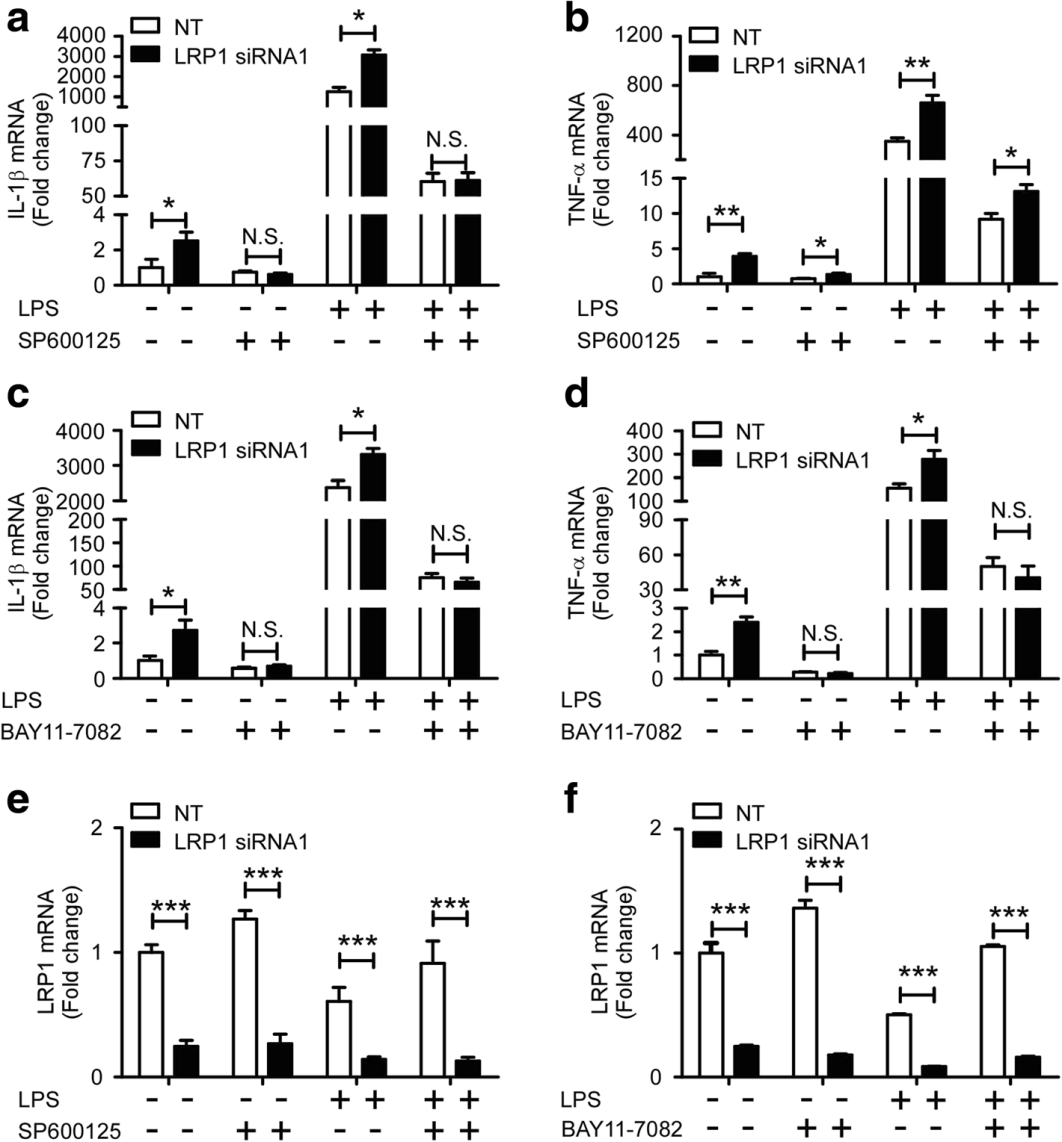
JNK and NF-κB inhibitors suppress the production of pro-inflammatory cytokines induced by LRP1 knockdown. **a**, **b** Mouse primary microglia were transiently transfected with non-targeting siRNA (NT) or LRP1-specific siRNA#1 for 48 h. Cells were stimulated with 100 ng/mL LPS or vehicle for 4 h in the presence or absence of 10 μM SP600125 (pretreated for 30 min). The qRT-PCR analysis was then performed to detect the expression levels of IL-1β (**a**) and TNF-α (**b**) (*n* = 3). β-Actin was used as an internal control. **c**, **d** Mouse primary microglia were transiently transfected with non-targeting siRNA (NT) or *Lrp1*-specific siRNA#1 for 48 h. The cells were then pretreated with 10 μM Bay 11-7082 for 30 min, followed by treatment with 100 ng/mL LPS or vehicle for 4 h. RNA was extracted and the relative mRNA levels of IL-1β (**c**) and TNF-α (**d**) were determined by qRT-PCR (*n* = 3). β-Actin was used as an internal control. Data were plotted as mean ± SEM and normalized to the corresponding control group. **e**, **f** Reduction of *Lrp1* mRNA on siRNA-mediated knockdown was verified by qRT-PCR. *p < 0.05; **p < 0.01; ***p < 0.001; *N.S.* not significant (one-way ANOVA with post hoc Tukey’s *t* test)

To further explore the molecular mechanism by which LRP1 down-regulation affects the pro-inflammatory responses, specific inhibitors for JNK (SP600125) and NF-κB (BAY11-7082) were used to block JNK and NF-κB activation. SP600125 is a cell-permeable small molecule that selectively inhibits all three JNK isoforms and prevents the phosphorylation of downstream JNK target c-Jun [39]. Our results showed that the mRNA level of IL-1β was increased upon LRP1 knockdown; however, the effect was abolished by pre-treatment with JNK inhibitor (Fig. 4a). The expression of TNF-α followed a similar trend though its level remained significantly higher in *Lrp1*-KD microglia than in control cells in the presence of JNK inhibitor (Fig. 4b). Furthermore, we tested the effect of BAY11-7082 which inhibits NF-κB pathway by reducing the level of phosphorylated IκBα [40]. Our results showed that the presence of NF-κB inhibitor eliminated the increased levels of IL-1β and TNF-α resulted from *Lrp1* down-regulation (Fig. 4c, d). Reduction of *Lrp1* mRNA level upon siRNA-mediated knockdown was confirmed in these experiments (Fig. 4e, f). Taken together, we concluded that LRP1 regulates the pro-inflammatory responses by modulating the activation of JNK and NF-κB signaling pathways.

### LRP1 expression in primary microglia is down-regulated by pro-inflammatory stimuli

**Fig. 5:**
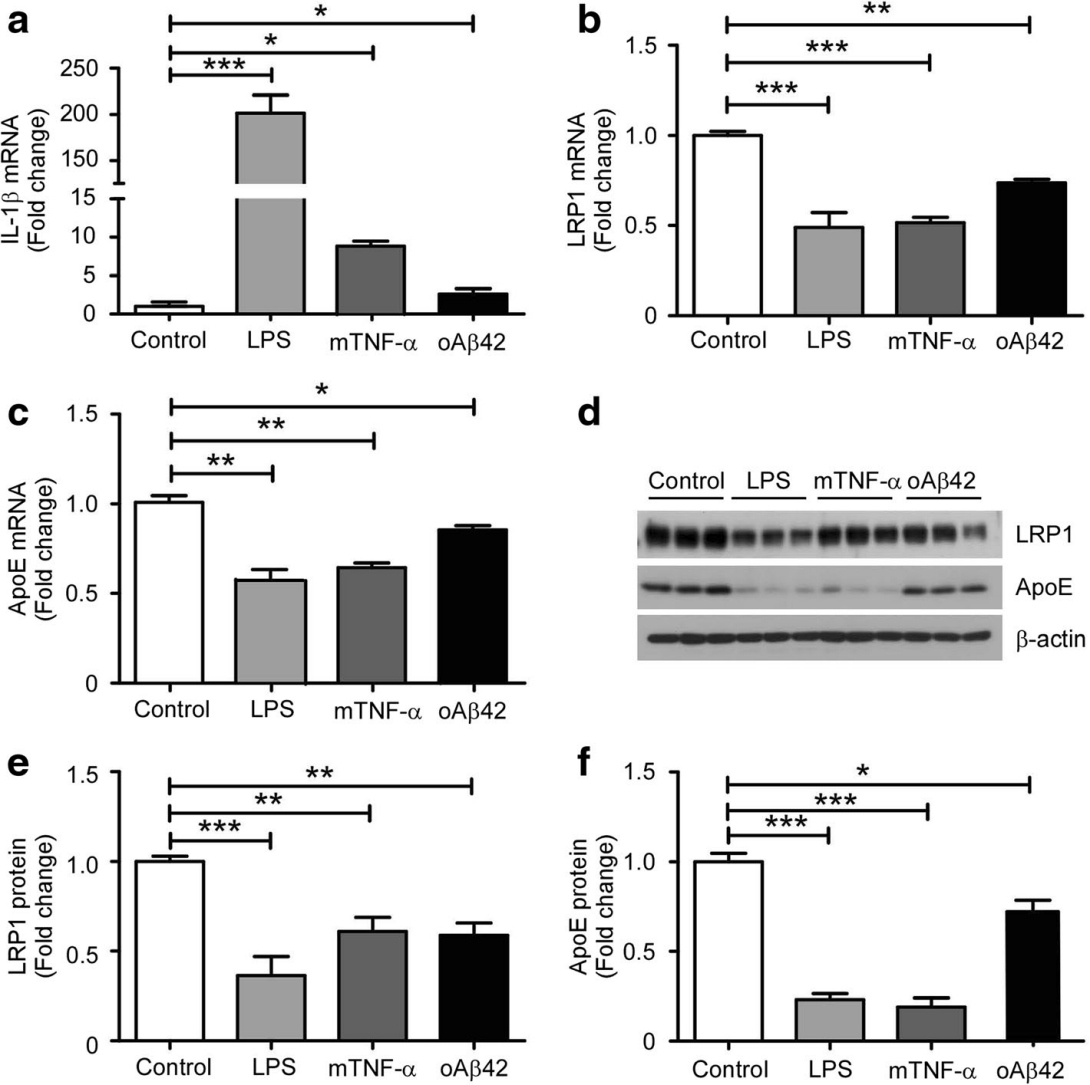
Pro-inflammatory cytokines and Aβ oligomers suppress the expression of LRP1 in microglia. **a**–**c** Primary microglia were cultured in the presence of either LPS (100 ng/ml), mouse TNF-α (100 ng/ml), or oligomeric Aβ (10 μM) for 24 h. RNA was extracted and the relative mRNA levels of *Il-1β* (**a**), *Lrp1* (**b**), and *Apoe* (**c**) were determined and quantified by qRT-PCR (*n* = 3). β-Actin was used as an internal control. **d**–**f** Cell lysates from the same treatments were collected, and the protein levels of LRP1 and apoE were analyzed by Western blot (**d**) and quantified (**e**, **f**). Data represent mean ± SEM and normalized to the corresponding control group. *p < 0.05; **p < 0.01; ***p < 0.001 (one-way ANOVA with post hoc Tukey’s *t* test)

Previous studies have shown that inflammatory mediators decrease LRP1 levels in macrophage [41, 42]. Since our data demonstrated an important role of LRP1 in the regulation of inflammatory responses within microglia, we therefore investigated the effects of pro-inflammatory stimuli (LPS, mouse TNF-α, and neurotoxic Aβ) on LRP1 expression. Microglial cells were treated with 100 ng/mL LPS, 50 ng/mL TNF-α, or 10 μM oligomeric Aβ42 for 24 h, respectively, then the mRNA and protein levels of LRP1 and various genes were examined. Consistent with previous reports, the mRNA levels of IL-1β were increased by these stimuli (Fig. 5a). Interestingly, both the mRNA and protein levels of LRP1 were down-regulated by the pro-inflammatory stimuli, including LPS, TNF-α, and oligomeric Aβ (Fig. 5b–d). Furthermore, the levels of apoE, which binds LRP1 as a ligand, were significantly down-regulated in the presence of these pro-inflammatory mediators (Fig. 5c, f). Our results indicate that various inflammatory mediators tightly regulate the expression of LRP1 in microglia.

### NF-κB inhibitor restores LRP1 expression down-regulated by LPS

**Fig. 6:**
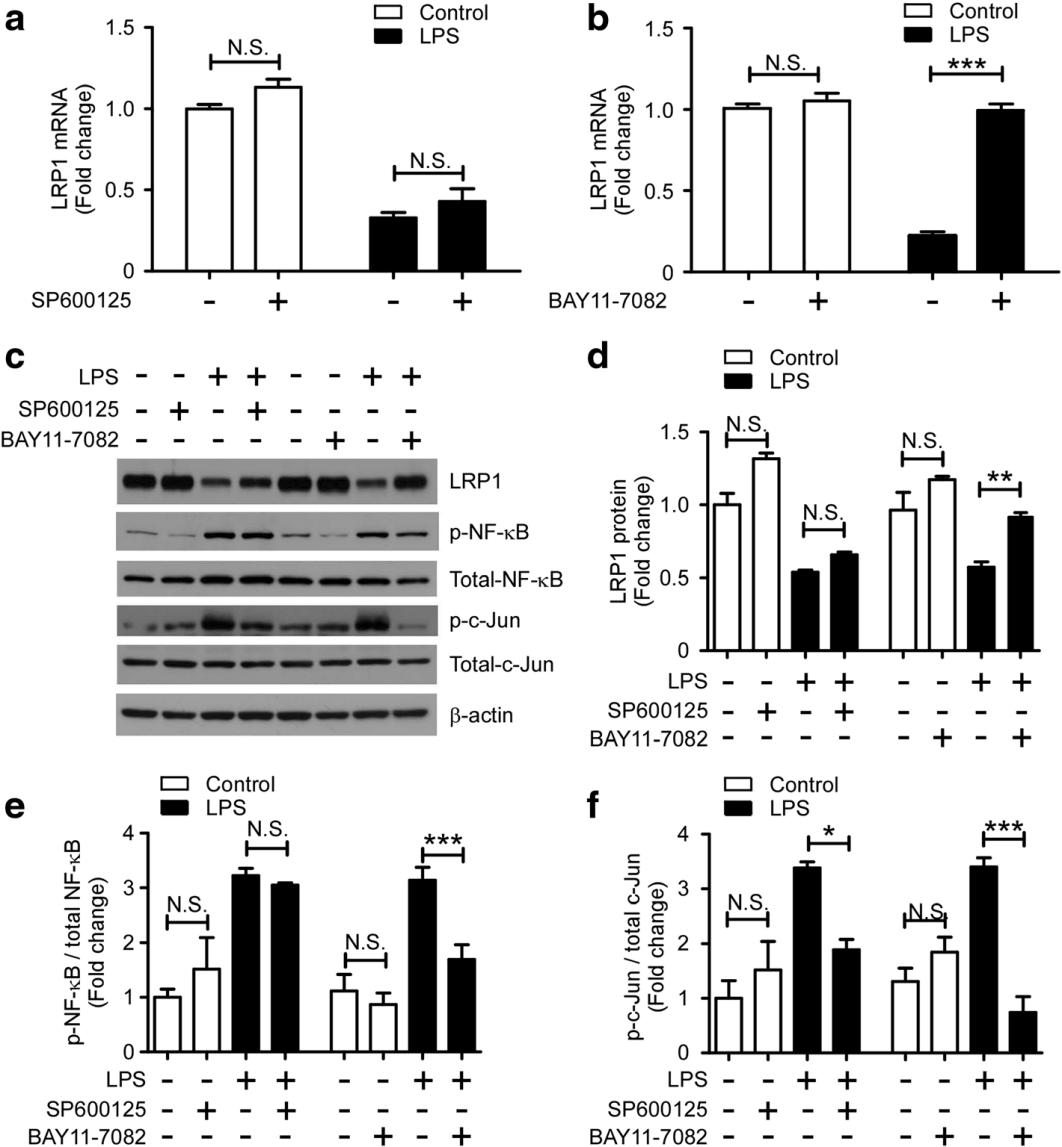
NF-κB inhibitor restores LRP1 expression suppressed by LPS. **a**–**b** Primary microglia were pretreated with 10 μM SP600125 or Bay 11-7082 for 30 min, followed by stimulation with LPS (100 ng/mL) or vehicle for 24 h. RNA was extracted, and the relative mRNA levels of *Lrp1* in microglia treated with SP600125 (**a**) and BAY11-7082 (**b**) were determined by qRT-PCR (*n* = 3). β-Actin was used as an internal control. Data were plotted as mean ± SEM ***p < 0.001; *N.S.* not significant (two-tailed Student’s *t* test). **c**–**f** The protein levels of LRP1, phospho-NF-κB, phospho-c-Jun, phospho-IκBα, total NF-κB, total c-Jun, and β-actin in cell lysates were examined by Western blot analysis (**c**) and quantified (**d**–**f**) (*n* = 3). Data represent mean ± SEM. ***p < 0.001; *N.S.* not significant (one-way ANOVA with post hoc Tukey’s *t* test)

Our results revealed that LRP1 modulates the LPS-mediated inflammatory response in microglia via JNK and NF-κB signaling pathways. In Fig. 4e, f, we noticed that LRP1 mRNA levels were significantly increased upon BAY11-7082 treatment for 4 h. We further examined whether these pathways were involved in the expression regulation of LRP1 by LPS. The mRNA level of *Lrp1* was similarly down-regulated by LPS in the presence or absence of JNK inhibitor for 24 h (Fig. 6a). However, the LPS-induced LRP1 down-regulated expression was restored by NF-κB inhibitor (Fig. 6b). Similarly, the protein level of LRP1 was decreased by LPS treatment, whereas this effect was reversed by NF-κB inhibitor, but not JNK inhibitor (Fig. 6c, d). In addition, we found that BAY11-7082 not only acted as a NF-κB inhibitor (Fig. 6e) but also significantly suppressed the LPS-induced c-Jun activation (Fig. 6f). Taken together, we conclude that the NF-κB signaling pathway downstream of LPS modulates the expression of LRP1 in microglia.

## Discussion

Neuroinflammation induced by microglial activation is an important pathological feature and an early event in the pathogenesis of AD. The predominant microglia phenotype is termed M1 state associated with a chronic neuroinflammatory environment accompanied by the increased and sustained release of pro-inflammatory mediators that modify AD progression [8, 43–45]. The important role of neuroinflammation in AD is also supported by the identification of AD risk genes including *TREM2* and *CD33* that predominantly expressed in microglia [10, 11, 46]. LRP1 is a widely studied receptor due to its involvement in multiple pathways in AD pathogenesis including modulation of Aβ clearance, lipid transport, and synaptic functions [22, 29, 47]. As the major immune cell type in the CNS, microglia have been reported to express higher levels of LRP1 transcript in the brain tissues of wild-type mice [48]. Several studies revealed that LRP1 might regulate microglial functions in CNS. The uptake of Aβ-coated yeast particles in microglia was suppressed by the presence of LRP1 ligands, indicating that LRP1 might regulate Aβ phagocytosis in microglia [49]. LRP1 was also shown to mediate phagocytosis of apoptotic cells by binding to cell surface calreticulin [50]. In addition, recent work by Chuang et al. demonstrated that in the brains of microglial *Lrp1* conditional knockout mice, microglia adopt a pro-inflammatory phenotype characterized by amoeboid morphology, indicating that LRP1 may regulate microglial activation in vivo. Of note, their work also suggests that ablation of LRP1 in microglia, not in macrophage, had a significant impact on the disease severity of multiple sclerosis [51]. However, the regulation and function of LRP1 as well as related signaling pathways in microglia remain to be elucidated. In the present study, we found that down-regulating LRP1 expression in microglia results in an increase of pro-inflammation cytokines. We also demonstrated that suppression of LRP1 expression or function in microglia leads to the activation of both JNK and NF-κB signaling pathways, suggesting that LRP1 in microglia directly regulates specific signaling pathways critical for inflammatory responses.

Previous studies have shown that LRP1 can directly regulate cellular signaling pathways via multiple mechanisms [52]. Here, we found that there was an aberrant JNK activation in LRP1-deficient microglia. Interestingly, the cytoplasmic domain of LRP1 (LRP1-ICD) binds JNK-interacting proteins (JIP-1 and JIP-2), which have been identified as modulators of the JNK signal transduction pathway [53]. It has also been demonstrated that overexpression of LRP1-ICD selectively prevents the activated JNK from translocating into the nucleus and, subsequently, nuclear transactivation of the JNK-dependent transcription factors, c-Jun and/or Elk-1 [54]. It is possible that LRP1 sequesters JNK by forming a complex with JIP, through which it regulates the JNK activation in microglia.

Consistent with the study by Chuang et al. in macrophage [51], our current study also found that LRP1 down-regulation in primary microglia not only leads to NF-κB activation in the absence of inflammatory stimuli but also enhances the LPS-induced NF-κB activity. Gaultier et al. has described a potential mechanism through which LRP1 may suppress NF-κB activation in mouse macrophage. Loss of LRP1 leads to increased surface expression of TNF-α receptor 1 (TNFR1) thus sensitizing the cell to the inflammatory signaling initiated by TNF-α. This results in an increased activation of NF-κB via phosphorylation and degradation of its inhibitory binding protein IκB [55]. Further studies are needed to understand the mechanism underlying LRP1-mediated NF-κB activation in microglia.

LRP1 regulates the metabolism of over 40 extracellular ligands; however, existing microglial *Lrp1* conditional knockout mouse model does not address the role of LRP1 as a receptor for diverse ligands [51]. Here we have explored the role of LRP1 antagonist RAP in microglial activation. We observed that RAP promotes the expression of pro-inflammatory cytokines and triggers JNK and NF-κB activation in microglia. Although the specific LRP1 ligands that mediate the inflammatory responses remain to be identified, our results suggest that RAP may inhibit the binding of ligands to LRP1 and therefore trigger microglial activation. Indeed, a previous study has shown that the LRP1 agonist attenuated the expression of pro-inflammatory mediators even in the presence of LPS, while the antagonist and LRP1 antibody that block its function had an opposite effect in macrophage [56]. Identifying specific LRP1 ligands that are involved in the regulation of microglial activation might provide a novel target for AD therapy.

LRP1 is highly expressed in the brain under normal physiological conditions, while it has been reported that the brains of AD patients had significantly lower LRP1 levels than those of age-matched controls. Moreover, higher LRP1 levels significantly correlate with later ages at onset of AD, while age and LRP1 expression in normal individuals appear to be inversely correlated [57]. To our knowledge, there are few reports regarding LRP1 function or the expression regulation of LRP1 in microglia during AD progression. Others and us have found that various stimuli (including LPS, TNF-α, and oligomeric Aβ42) down-regulate the microglial expression of LRP1 and apoE but increase the expression of pro-inflammatory cytokines, indicating that the expression of LRP1 and its ligands is essential for modulating glial activation [58, 59]. Indeed, it has been shown that LRP1 ligand apoE modulates microglial inflammation through LRP1 [53]. Together, LRP1 expression may be either down-regulated in glial cells due to neuroinflammation or suppressed in neurons due to post-synaptic damages in AD. Further studies are needed to clarify the temporal and spatial regulation of LRP1 expression during AD progression.

Since both JNK and NF-κB pathways are responsible for microglial activation in LRP1-deficient microglia or in the presence of LRP1 antagonist, we further investigated whether blocking these pathways could suppress the production of pro-inflammatory cytokines induced by LRP1 down-regulation in microglia. We found that JNK inhibitor (SP600125) blocks the increase of IL-1β in *Lrp1*-KD microglia; however, it failed to restore the level of TNF-α. Interestingly, the higher levels of pro-inflammatory cytokines seen in *Lrp1*-KD microglia were significantly suppressed by NF-κB inhibitor (BAY11-7082). Our results further showed that NF-κB inhibitor rescues LPS down-regulated LRP1 expression. As JNK pathway was also repressed by BAY11-7082, this inhibitor might block both JNK and NF-κB pathways. Thus, the precise mechanism requires further investigation using molecular and genetic approaches in addition to the pharmacological inhibitors used in this study.

As central signaling pathways of neuroinflammation, the activation of JNK and NF-κB is involved in several pathophysiological processes of AD. In the brain of AD patients, activated JNK and NF-κB were found predominantly in neurons and glial cells in areas surrounding Aβ plaque [60–65]. Several studies have shown that JNK and NF-κB inhibitors are effective in slowing down disease progression. Some nonsteroidal anti-inflammatory drugs (NSAIDs) have a direct effect on NF-κB activity, which also leads to a decrease in Aβ production. Flurbiprofen and indomethacin, which target NF-κB, have been shown to effectively reduce the amyloid plaque load in AD mouse models [66, 67]. JNK inhibition could promote the expression of apoE in microglia to reduce inflammation [68]. Moreover, inhibition of JNK activation by chronic treatment of SP600125 markedly reduces multiple pathological features and ameliorates cognitive deficits in APPswe/PS1dE9 mice [69]. Based on our current findings, BAY11-7082 may be a promising anti-inflammatory inhibitor targeting LRP1 for the treatment of AD.

## Conclusions

In summary, we demonstrate that LRP1 suppresses microglial activation by modulating JNK and NF-κB signaling pathways. Down-regulation of LRP1 levels and the increased pro-inflammatory signaling may result in a vicious cycle, in which the two events synergistically promote microglial activation. Restoration of LRP1 expression in microglia may serve as a novel therapeutic approach to combat microglial dysfunction associated with chronic inflammation in neurodegenerative diseases including AD. Further studies should be carried out in AD mouse models deficient of microglial LRP1 to better understand the specific functions of LRP1 in microglia in the presence of AD-related pathologies.

## Abbreviations

apoE: Apolipoprotein E
Aβ: Amyloid-β
CNS: Central nervous system
ELISA: Enzyme-linked immunosorbent assay
ICD: Intracytoplasmic domain
IL-1β: Interleukin-1β
JIPs: JNK-interacting proteins
JNK: c-Jun N-terminal kinase
LPS: Lipopolysaccharide
LRP1: Low-density lipoprotein receptor-related protein 1
*Lrp1-KD*: *Lrp1*-knockdown
MAPK: Mitogen-activated protein kinase
NF-κB: Nuclear factor-κB
NSAIDs: Nonsteroidal anti-inflammatory drugs
qRT-PCR: Quantitative real-time PCR
RAP: Receptor-associated protein
TLR4: Toll-like receptor 4
TNFR1: Tumor necrosis factor-α receptor 1
TNF-α: Tumor necrosis factor-α
